# The complete chloroplast genome sequence of a cultivar of Chrysanthemum, *Chrysanthemum* × *morifolium* ‘Hangbaiju’ (Asteraceae)

**DOI:** 10.1080/23802359.2024.2334014

**Published:** 2024-03-25

**Authors:** Zhangliang Yao, Jiashun Miao, Weidong Xu, Qiang Lu

**Affiliations:** a Institute of Eco-Environmental Sciences, Jiaxing Academy of Agricultural Sciences, Zhejiang, China; b Okinawa Institute of Science and Technology Graduate University, Okinawa, Japan

**Keywords:** Asteraceae, chloroplast genome, *Chrysanthemum*, Hangbaiju, phylogenetic analysis

## Abstract

*Chrysanthemum* × *morifolium* Ramat 1792 cultivar ‘Hangbaiju’, also known as ‘Hangzhou White Chrysanthemum’, originates from Tongxiang City, Zhejiang Province, China. It is celebrated as one of Zhejiang’s ‘eight flavors’. In this study, we reported the complete chloroplast genome of *Chrysanthemum* × *morifolium* cultivar ‘Hangbaiju’. The genome has a circular structure of 151,110 bp containing a large single-copy region (LSC) of 82,851 bp, a small copy region (SSC) of 18,351 bp, and two inverted repeats (IR) of 24,936 and 24,972 bp in length. It comprises 128 genes: 85 protein-coding gene, 8 ribosomal RNA (rRNA) genes, and 35 transfer RNA (tRNA) genes. Phylogenetic analysis, based on complete chloroplast genomes, demonstrates that *Chrysanthemum* × *morifolium* ‘Hangbaiju’ shares a close genetic cluster with *Chrysanthemum* × *morifolium* ‘Fubaiju’ (MT1919691.1). Notably, ‘Fubaiju’ was introduced to Macheng, Hubei Province from Tongxiang in 1968 according to public information. The chloroplast genome data, coupled with morphological and historical records, strongly suggest that they are the same variety known by different names based on their cultivation locations.

## Introduction

**Figure 1.**
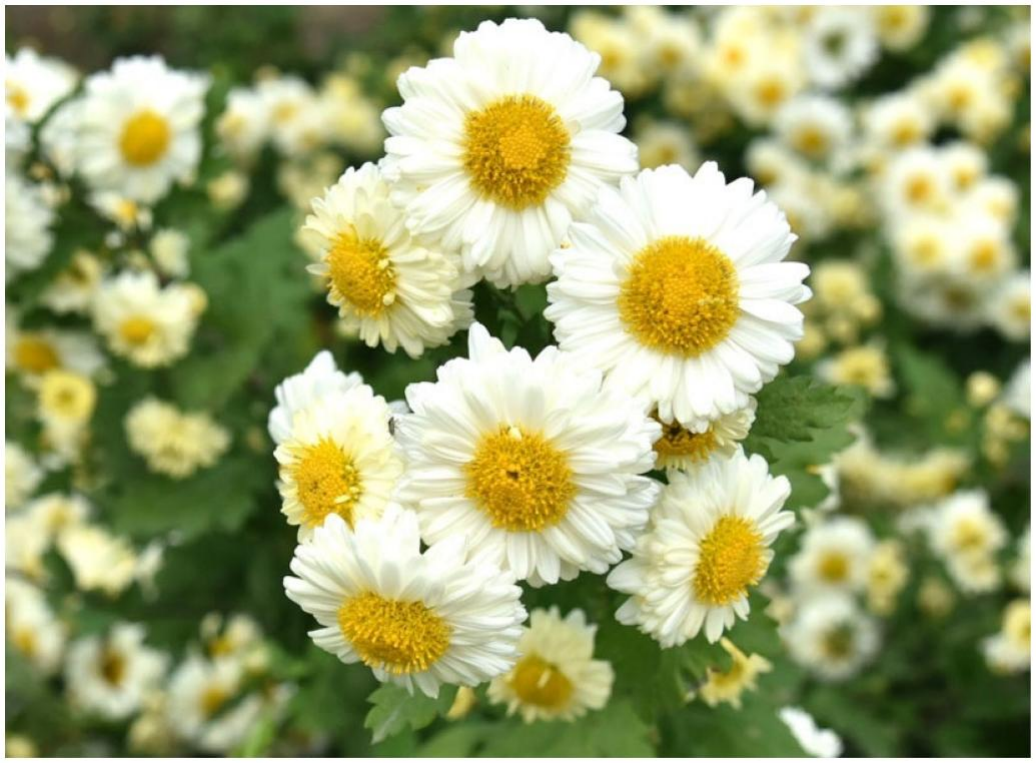
The flowers of *Chrysanthemum* × *morifolium* ‘Hangbaiju’. The capitulum inflorescence is solitary or several clustered at the top of the stem and branches, with several layers of tongue-shaped flowers located on the periphery, which are whitish in color. The photo was taken by author Zhangliang Yao at the sampling site (30.6451 N and 120.4799E).

*Chrysanthemum* × *morifolium* Ramat cultivar ‘Hangbaiju’ is a perennial herbaceous plant of the *Chrysanthemum* genus in the Asteraceae family. It has an erect stem covered with soft hairs. The leaves are arranged alternately, with short petioles, and are ovate to lanceolate in shape. And they are pinnately lobed or deeply lobed, with a wedge-shaped base and covered with white short soft hairs on the back. The capitulum inflorescence is solitary or several together at the top of the stem and branches (Figure 1). The ray florets are in several layers and are white or similar in color (Figure 1). The flowering period of Hangbaiju is in October. Hangbaiju has a rich history and is prized for its use in making floral tea, particularly the famous ‘Hangzhou White Chrysanthemum Tea’ for its whitish tongue-shaped flowers (Figure 1). It is renowned for its unique fragrance and flavor, and is believed to have various medicinal properties, such as clearing heat, detoxifying, reducing internal heat, and soothing the liver (Long et al. 2023). In this study, we report the complete chloroplast genomes of *Chrysanthemum* × *morifolium* ‘Hangbaiju’, which could be used for the cultivar identification.

## Materials and methods

Fresh leaves of *Chrysanthemum* × *morifolium* ‘Hangbaiju’ were sampled in Tongxiang, City, Zhejiang Province, China (coordinates: 30.6451 N, 120.4799E). The collected specimen. labeled TXHBJ001, is now preserved at Institute of Eco-Environmental Sciences, Jiaxing Academy of Agricultural Sciences (https://www.jxnky.com) under the stewardship of Zhangliang Yao (zlyao513@126.com). We extracted whole genomic DNA from these fresh leaves utilizing the CTAB method (Doyle and Doyle 1987). The extracted whole genomic DNA was then used to prepare a sequencing library, which was sequenced on Illumina NovaSeq 6000 platform (Illumina Inc., San Deigo, CA, USA) with a paired-end sequencing length of 150 bp. Raw sequencing data were filtered and trimmed using fastp (version 0.23.2) (Chen et al. 2018) following default settings. The complete chloroplast genome was then assembled with processed data by GetOrangelle (version 1.7.7.0) (Jin et al. 2020). Annotation of chloroplast genome was performed with CpGAVAS2 software (Shi et al. 2019). The Geneious Prime (Kearse et al. 2012) was used to correct these annotations with problems. Visualization of the chloroplast genome was achieved with OrganellarGenomeDRAW (OGDRAW) (version 1.3.1) (Greiner et al. 2019) and CPGView (www.1kmpg.cn/cpgview/; Liu et al. 2023). We identify microsatellite in chloroplast genome using MISA (v2.1) (Beier et al. 2017) with following parameters: ‘1-10 2-6 3-5 4-5 5-5 6-5′. Tandem repeats were identified using Tandem Repeats Finder (version 4.09) (Benson 1999) with following parameters: ‘2 7 7 80 10 50 500 -f -d -m’. Sequencing depth and coverage plot of the assembled chloroplast genome was assessed by aligning raw reads to the assembled chloroplast genome using BWA-mem (version v0.7.17) (Li and Durbin 2009). Base depth was calculated by samtools depth (version 1.16.1) (Li et al. 2009) with the alignment file.

In addition to the chloroplast genome of *C.* × *morifolium* ‘Hangbaiju’ assembled in current study, we downloaded 22 other species/varieties within the family of Asteraceae and Rubiaceae from NCBI. Multiple sequence alignment was performed using with MAFFT (version 7) (Katoh and Srandley 2013) with auto strategy. Subsequently, these aligned sequences were used to construct the Maximum Likelihood tree with IQ-TREE (version 2.0.7) (Nguyen et al. 2015) with 1,000 bootstrap replicates.

## Results and discussion

**Figure 2.**
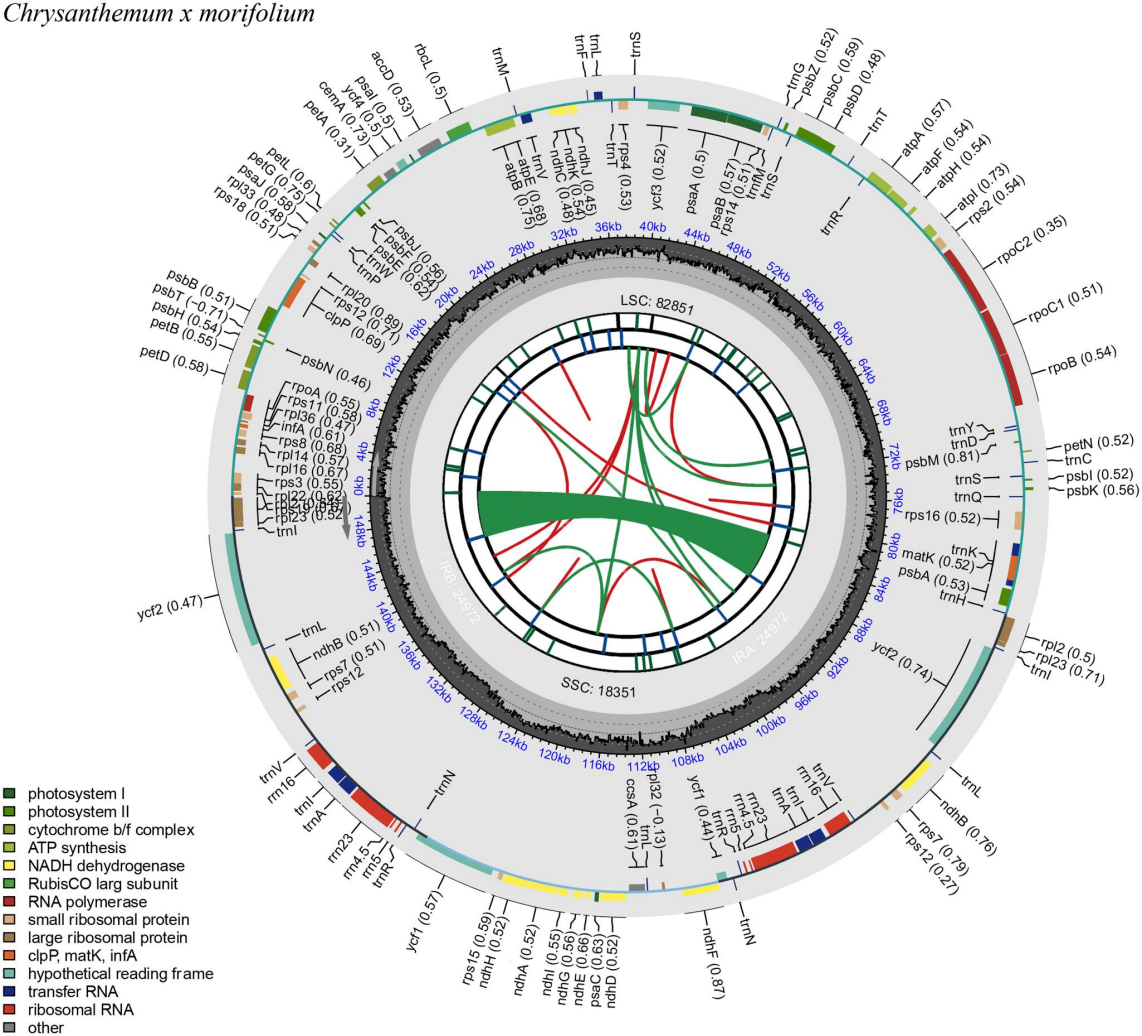
Chloroplast genome map of *Chrysanthemum* × *morifolium* ‘Hangbaiju’ visualized by CPGview. The illustration of six tracks (from center to outward): first track: direct and palindromic repeats. These are linked by red and green arcs, respectively. Second track: long tandem repeats with short blue bars. Third track: short tandem repeats or microsatellites using colored bars. The color coding is: black: complex repeat (c), green: repeat unit of 1, yellow: repeat unit of 2, purple: repeat unit of 3, blue: repeat unit of 4, orange: repeat unit of 5, red: repeat unit of 6. Fourth track: the quadripartite structure (LSC, SSC, IRA, and IRB). Fifth track: GC content. Sixth track: genes. Each gene displays its optional codon usage bias in parentheses. The genes are color-coded based on their functional classification, with the key in the bottom left corner. The transcription directions for the inner and outer genes are clockwise and anticlockwise, respectively.

The complete circular chloroplast genome of *C.* × *morifolium* ‘Hangbaiju’ (GenBank accession no. OR486294.1) spans 151,110 base pairs (bp). It is organized into a large single-copy region (LSC) of 82,851 bp, a small copy region (SSC) of 18,351 bp, and two inverted repeats (IRs) each measuring 24,936 and 24,972 bp, respectively. The chloroplast genome was assembled from Illumina short read sequences and the sequencing depth of the bases in the assembled genome ranges from 32× to 370×, with a mean depth of 174.62× and a standard deviation of 29.53× (Figure S1). The ‘Hangbaiju’ chloroplast genome is composed of 128 genes, of which 108 are unique. This includes 85 protein-coding gene (78 unique), 8 ribosomal RNA (rRNA) genes (4 unique), and 35 transfer RNA (tRNA) genes (26 unique). Notably, it contains 19 genes with a single intron, and two genes with two introns (Table S1 and S2). The cis-splicing genes and the trans-splicing gene rps12 are shown in supplementary Figure S2 and S3. The total length of the protein-coding sequence (CDS) within this chloroplast genome of is 78,609 bp. The overall GC content of entire ‘Hangbaiju’ chloroplast is approximately 37.45%, with the LSC and SSC regions having 35.54% and 30.78%, respectively (Figure 2). It is notably that IRa region has the highest GC content (43.07%) and IRb has the second high GC content (36.70%) (Figure 2). For microsatellite analysis, we utilized MISA (v2.1) (Beier et al. 2017), identifying 41 SSR-makers within the ‘Hangbaiju’ chloroplast genome (Table S3). Furthermore, the Tandem Repeats Finder (version 4.09) (Benson 1999) was used to identify the tandem repeats in ‘Hangbaiju’ chloroplast, and 31 tandem repeats were found (Table S4). The variability and high mutation rates of microsatellites and tandem repeats in chloroplast genome make them excellent markers for studying genetic diversity, population structure, and evolutionary relationships among plant species. This can be particularly useful in conservation biology for identifying genetically distinct populations or in agriculture for crop breeding programs.

**Figure 3.**
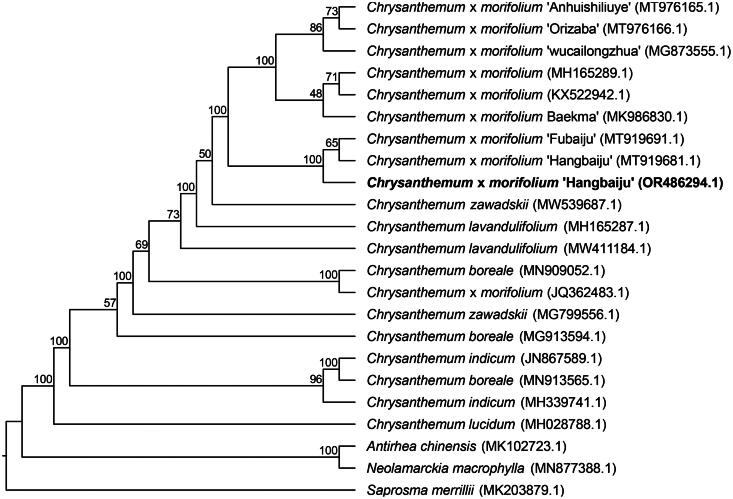
Phylogenetic analysis of *Chrysanthemum* × *morifolium* ‘Hangbaiju’ OR486294.1 and other 22 species/varieties within family Asteraceae and Rubiaceae. The following chloroplast genome sequences were used: *Chrysanthemum* × *morifolium* ‘Hangbaiju’ MT919681.1, *Chrysanthemum* × *morifolium* KX522942.1 (Hongmei et al. 2021), *Chrysanthemum* × *morifolium* MH165289.1 (Ma et al. 2020), *Chrysanthemum* × *morifolium* ‘Baekma’ MK986830.1 (Tyagi et al. 2019), *Chrysanthemum* × *morifolium* ‘Anhuishiliuye’ MT976165.1 (Xia et al. 2021), *Chrysanthemum* × *morifolium* ‘Orizaba’ MT976166.1 (Xia et al. 2021), *Chrysanthemum* × *morifolium* ‘wucailongzhua’ MG873555.1 (Ma et al. 2020), *Chrysanthemum* × *morifolium* ‘Fubaiju’ MT919691.1 (Zeng et al. 2021), *Chrysanthemum lavandulifolium* MH165287.1 (Ma et al. 2020) *Chrysanthemum* × *zawadskii* MW539687.1 (Baek et al. 2021), *Chrysanthemum lavandulifolium* MW411184.1, *Chrysanthemum* × *morifolium* JQ362483.1, *Chrysanthemum boreale* MN909052.1 (Tyagi et al. 2020), *Chrysanthemum zawadskii* MG799556.1 (Ma et al. 2020), *Chrysanthemun boreale* MG913594.1 (Won et al. 2018), *Chrysanthemum indicum* JN867589.1, *Chrysanthemum boreale* MH913565.1, *Chrysanthemum indicum* MH339741.1 (Ma et al. 2020), *Chrysanthemum lucidum* MH028788.1, *Antirhea chinensis* MK102723.1 (Fan et al. 2019), *Neolamarckia macrophylla* MN877388.1, *Saprosma merrillii* MK203879.1 (Zhu et al. 2019). Maximum likelihood tree was reconstructed based on complete chloroplast genome sequences with 1,000 bootstrap replicates. The bootstrap values were indicated at nodes. The 21 chloroplast genome sequences were obtained from NCBI GenBank databases (accession numbers have marked on the figure) and related literatures were listed after the GenBank accession numbers.

To determine the phylogenetic position of ‘Hangbaiju’, we generated a Maximum Likelihood (ML) tree by utilizing the complete chloroplast genome of ‘Hangbaiju’ and 21 other species/varieties within the family of Asteraceae and Rubiaceae. The phylogenetic analysis showed *C.* × *morifolium* ‘Hangbaiju’ was clustered with *C.* × *morifolium* ‘Fubaiju’ (accession number: MT919691.1) (Figure 3). Given the introduction history of ‘Fubaiju’ from Tongxiang to Macheng City in 1968 and the morphological similarities between ‘Fubaiju’ and ‘Hangbaiju’, we infer that these two are indeed the same variety. Hangbaiju (Hangzhou White Chrysanthemum) and Fubaiju (Fuzhou White Chrysanthemum), although the same variety of tea, are differentiated based on their geographical names. This practice is common in many agricultural products, especially in the realms of tea, wine, and coffee. Many regions are renowned for their unique climate and soil conditions, which can affect the growth and quality of plants. These conditions can impart unique flavors and characteristics to the same variety grown in different areas. By using place names, producers can emphasize the uniqueness and origin of their products.

This research advances our understanding of the genetic diversity within the *Chrysanthemum* genus and contributes to the broader field of plant genomics and taxonomy. The comprehensive analysis of the chloroplast genome of ‘Hangbaiju’ not only offers insights into its phylogenetic position but also underscores the significance of genomic studies in confirming the identity and history of plant cultivars. Our findings highlight the potential of chloroplast genome analysis as a reliable tool for plant identification and classification, particularly in cases where morphological characteristics and historical records may be ambiguous or incomplete.

## Conclusions

In conclusion, our comprehensive chloroplast genomic analysis of *Chrysanthemum* × *morifolium* ‘Hangbaiju’ not only clarifies its relationship with ‘Fubaiju’ but also contributes valuable genetic information to the field of *Chrysanthemum* taxonomy.

## Supplementary Material

Supplemental Material

## Data Availability

The genome sequence data that support the findings of this study are openly available in GenBank of NCBI at https://www.ncbi.nlm.nih.gov/ under the accession no. OR486294.1. The associated ‘BioProject’, ‘SRA’ and ‘Bio-Sample’ numbers are PRJNA1026330, SRX22038282, and SAMN37737287 respectively.
