# Supplementary material for: The complete chloroplast genome sequence of a cultivar of Chrysanthemum, Chrysanthemum × morifolium ‘Hangbaiju’ (Asteraceae)
Source: Mitochondrial DNA B Resour. 2024 Mar 25;9(3):403–7. doi: 10.1080/23802359.2024.2334014 (PMC10967664; doi:10.1080/23802359.2024.2334014)
Supplement: Supplemental Material [file TMDN_A_2334014_SM7330.docx]

**Supplementary materials**

**The complete chloroplast genome sequence of a cultivar of *Chrysanthemum*, *chrysanthemum*** × ***morifolium* ‘Hangbaiju’ (Asteraceae)**

Zhangliang Yao^a*^, Jiashun Miao^b*^, Weidong Xu^a^, Qiang Lu^a^

^a^Institute of Eco-Environmental Sciences, Jiaxing Academy of Agricultural Sciences, Zhejiang, China; ^b^Okinawa Institute of Science and Technology Graduate University, Okinawa, Japan;

Correspondence: Jiashun Miao, [miaojiashun@126.com](mailto:miaojiashun@126.com), Okinawa Institute of Science and Technology Graduate University, Okinawa, Japan; Qiang Lu, [jxluqiang@163.com](mailto:jxluqiang@163.com), Institute of Eco-Environmental Sciences, Jiaxing Academy of Agricultural Sciences, Zhejiang, China.

*These authors contributed equally to this study and share the first authorship.

**Contents:**

**Figure S1.** Sequence depth and coverage map of *Chrysanthemum morifolium* cultivar ‘Hangbaiju’

**Figure S2.** The cis-splicing genes existed in *Chrysanthemum morifolium* cultivar ‘Hangbaiju chloroplast genome.

**Figure S3.** Schematic map of the trans-splicing gene rps12 in *Chrysanthemum morifolium* cultivar ‘Hangbaiju chloroplast genome.

**Table S1.** List of one intron containing genes in *Chrysanthemum morifolium* cultivar ‘Hangbaiju chloroplast genome.

**Table S2.** List of two introns containing genes in *Chrysanthemum morifolium* cultivar ‘Hangbaiju chloroplast genome.

**Table S3.** Simple Sequence Repeats (SSR)/Microsatelite identified in the chloroplast genome of *Chrysanthemum morifolium* cultivar ‘Hangbaiju’ with MISA (v2.1).

**Table S4.** Tandem repeats identified in the chloroplast genome of *Chrysanthemum morifolium* cultivar ‘Hangbaiju’ with TRF (version 4.09).

**Figure S1.** Sequence depth and coverage map of *Chrysanthemum morifolium* cultivar ‘Hangbaiju’

**Figure S2.** The cis-splicing genes existed in *Chrysanthemum morifolium* cultivar ‘Hangbaiju chloroplast genome.

**Figure S3.** Schematic map of the trans-splicing gene rps12 in *Chrysanthemum morifolium* cultivar ‘Hangbaiju chloroplast genome. It has three unique exons. Two of them are duplicated as they are in the IR regions.

**Table S1.** List of one intron containing genes in *Chrysanthemum morifolium* cultivar ‘Hangbaiju chloroplast genome.

| Genes | Exon1 | Exon2 |
| --- | --- | --- |
| *pet*D | [7617:7624](-) | [6467:6941](-) |
| *pet*B | [9202:9207](-) | [7813:8454](-) |
| *rps*12 | [14280:14393](+) | [137128:137370](+) |
| *rps*12_copy2 | [14280:14393](+) | [96556:96798](-) |
| *atp*F | [56013:56157](-) | [54904:55313](-) |
| *rpo*C1 | [66455:66886](-) | [64085:65722](-) |
| *rps*16 | [76527:76566](+) | [77449:77633](+) |
| *rpl*2 | [84066:84456](-) | [82967:83400](-) |
| *ndh*B | [94411:95189](-) | [92987:93743](-) |
| *ndh*A | [119172:119718](-) | [117569:118107](-) |
| *ndh*B_copy2 | [138737:139515](+) | [140183:140939](+) |
| *rpl*2_copy2 | [149506:149896](+) | [150562:150995](+) |
| *trn*V-UAC | [31124:31161](+) | [31734:31770](+) |
| *trn*L-UAA | [36199:36235](-) | [35725:35774](-) |
| *trn*K-UUU | [78480:78516](+) | [81097:81126](+) |
| *trn*I-GAU | [100710:100751](+) | [101528:101562](+) |
| *trn*A-UGC | [101627:101664](+) | [102477:102511](+) |
| *trn*A-UGC_copy2 | [132262:132299](-) | [131415:131449](-) |
| *trn*I-GAU_copy2 | [133175:133216](-) | [132364:132398](-) |

**Table S2.** List of two introns containing genes in *Chrysanthemum morifolium* cultivar ‘Hangbaiju chloroplast genome.

| Genes | Exon1 | Exon2 | Exon3 |
| --- | --- | --- | --- |
| *clp*P | [12127:12197](+) | [12995:13288](+) | [13901:14126](+) |
| *ycf*3 | [39000:39123](+) | [39835:40064](+) | [40808:40957](+) |

**Table S3.** Simple Sequence Repeats (SSR)/Microsatelite identified in the chloroplast genome of *Chrysanthemum morifolium* cultivar ‘Hangbaiju’ with MISA (v2.1).

| No.. | Type | SSR | size | start | end |
| --- | --- | --- | --- | --- | --- |
| 1 | p1 | (A)11 | 11 | 3109 | 3119 |
| 2 | p1 | (A)16 | 16 | 3609 | 3624 |
| 3 | p1 | (A)10 | 10 | 6033 | 6042 |
| 4 | p1 | (A)15 | 15 | 13541 | 13555 |
| 5 | p1 | (T)10 | 10 | 15645 | 15654 |
| 6 | p3 | (ATA)5 | 15 | 18680 | 18694 |
| 7 | p1 | (A)15 | 15 | 20749 | 20763 |
| 8 | p1 | (T)10 | 10 | 22940 | 22949 |
| 9 | p1 | (A)10 | 10 | 28430 | 28439 |
| 10 | p1 | (A)10 | 10 | 30827 | 30836 |
| 11 | p1 | (A)11 | 11 | 32623 | 32633 |
| 12 | c | (T)13….. (AT)6 | 109 | 36532 | 36640 |
| 13 | p1 | (A)10 | 10 | 39186 | 39195 |
| 14 | c | (T)17ag(A)10 | 29 | 41210 | 41238 |
| 15 | p1 | (T)11 | 11 | 47295 | 47305 |
| 16 | p1 | (A)10 | 10 | 48161 | 48170 |
| 17 | p1 | (A)14 | 14 | 51841 | 51854 |
| 18 | p1 | (T)10 | 10 | 53047 | 53056 |
| 19 | p1 | (A)10 | 10 | 53264 | 53273 |
| 20 | p1 | (A)12 | 12 | 54856 | 54867 |
| 21 | p1 | (A)10 | 10 | 56785 | 56794 |
| 22 | p1 | (A)10 | 10 | 57873 | 57882 |
| 23 | p1 | (A)12 | 12 | 59604 | 59615 |
| 24 | p1 | (T)10 | 10 | 64427 | 64436 |
| 25 | p1 | (T)10 | 10 | 64853 | 64862 |
| 26 | p1 | (T)10 | 10 | 69474 | 69483 |
| 27 | p1 | (A)21 | 21 | 70159 | 70179 |
| 28 | p1 | (A)10 | 10 | 72175 | 72184 |
| 29 | p1 | (A)15 | 15 | 80891 | 80905 |
| 30 | p1 | (T)10 | 10 | 82924 | 82933 |
| 31 | p1 | (T)10 | 10 | 101180 | 101189 |
| 32 | p1 | (A)10 | 10 | 106083 | 106092 |
| 33 | p1 | (T)10 | 10 | 106550 | 106559 |
| 34 | p1 | (T)10 | 10 | 110073 | 110082 |
| 35 | p1 | (A)10 | 10 | 110976 | 110985 |
| 36 | p1 | (T)12 | 12 | 112015 | 112026 |
| 37 | p1 | (A)12 | 12 | 124078 | 124089 |
| 38 | p1 | (A)10 | 10 | 127367 | 127376 |
| 39 | p1 | (T)10 | 10 | 127834 | 127843 |
| 40 | p1 | (A)10 | 10 | 132737 | 132746 |
| 41 | p1 | (A)10 | 10 | 151029 | 151038 |

**Table S4.** Tandem repeats identified in the chloroplast genome of *Chrysanthemum morifolium* cultivar ‘Hangbaiju’ with TRF (version 4.09).

| Position | Period size | Copy number | Consensus size | Percent Matches | Percent Indels |
| --- | --- | --- | --- | --- | --- |
| 2361-2391 | 15 | 2.1 | 15 | 100 | 0 |
| 6353-6380 | 14 | 2 | 14 | 100 | 0 |
| 16021-16061 | 21 | 2 | 21 | 95 | 0 |
| 17462-17497 | 17 | 2.1 | 17 | 100 | 0 |
| 18643-18698 | 16 | 3.2 | 16 | 73 | 22 |
| 18652-18708 | 29 | 2 | 28 | 93 | 3 |
| 18643-18700 | 23 | 2.6 | 23 | 71 | 13 |
| 26569-26618 | 24 | 2.1 | 24 | 100 | 0 |
| 31054-31088 | 15 | 2.3 | 15 | 90 | 0 |
| 32506-32550 | 17 | 2.6 | 17 | 83 | 10 |
| 35123-35158 | 15 | 2.4 | 15 | 86 | 13 |
| 36955-36988 | 17 | 2 | 17 | 100 | 0 |
| 47718-47742 | 13 | 1.9 | 13 | 100 | 0 |
| 52685-52727 | 22 | 2 | 22 | 90 | 4 |
| 57753-57777 | 12 | 2.1 | 12 | 100 | 0 |
| 72087-72118 | 16 | 2 | 16 | 100 | 0 |
| 77772-77819 | 15 | 3.3 | 15 | 91 | 2 |
| 80801-80843 | 21 | 2 | 21 | 100 | 0 |
| 80848-80872 | 13 | 1.9 | 13 | 100 | 0 |
| 90070-90126 | 18 | 3.4 | 17 | 83 | 11 |
| 90081-90126 | 9 | 5.1 | 9 | 78 | 0 |
| 90080-90139 | 18 | 3.3 | 18 | 95 | 0 |
| 97712-97736 | 10 | 2.5 | 10 | 100 | 0 |
| 105723-105784 | 32 | 1.9 | 32 | 96 | 0 |
| 107793-107820 | 14 | 2 | 14 | 100 | 0 |
| 110616-110645 | 15 | 2 | 15 | 100 | 0 |
| 116689-116734 | 23 | 2 | 23 | 100 | 0 |
| 128142-128203 | 32 | 1.9 | 32 | 96 | 0 |
| 136190-136214 | 10 | 2.5 | 10 | 100 | 0 |
| 143787-143882 | 18 | 5.3 | 18 | 97 | 0 |
| 143854-143892 | 18 | 2.4 | 16 | 87 | 12 |
